# Elution study of acrylic monomers from orthodontic materials using high performance liquid chromatography (HPLC)

**DOI:** 10.1007/s00056-021-00292-4

**Published:** 2021-04-14

**Authors:** B. J. Kux, L. M. Bacigalupo, A. Scriba, M. Emmrich, P.-G. Jost-Brinkmann

**Affiliations:** Institute of Dental, Oral and Maxillary Medicine, Department of Orthodontics, Dentofacial Orthopedics and Pedodontics, Charité—Universitätsmedizin Berlin, Corporate Member of Freie Universität Berlin and Humboldt-Universität zu Berlin, Aßmannshauser Str. 4–6, 14197 Berlin, Germany

**Keywords:** Bisphenol A‑glycidyl dimethacrylate, Triethylene glycol dimethacrylate, Urethane dimethacrylate, Adhesives, Orthodontic appliances, fixed, Bisphenol-A-Glycidyl-Dimethacrylat, Triethylenglykol-Dimethacrylat, Urethan-Dimethacrylat, Klebstoffe, Festsitzende kieferorthopädische Apparaturen

## Abstract

**Purpose:**

Main goal of the study was the identification and quantitative analysis of monomer elution from materials commonly used in fixed orthodontic therapy. Studies have shown severe health effects of monomers including cytotoxic, allergenic or mutagenic potential and endocrine changes. This in vitro study focusses primarily on five resins which are usually processed intraorally and remain in the oral cavity long-term.

**Methods:**

We tested the elution of monomers from specimens (7.5 mm × 1.5 mm) immersed in artificial saliva at body temperature (37 °C) for 30 min to 5 weeks. The used method is in accordance with DIN EN ISO 10993-13. The five tested materials were BrackFix® (Voco GmbH, Cuxhaven, Germany), Triad®Gel (DeguDent GmbH, Hanau, Germany), and Transbond™ XT, LR and Plus (3M Unitek, Monrovia, CA, USA). All aliquots were analyzed using high performance liquid chromatography (HPLC). Data were statistically analyzed.

**Results:**

All five analyzed materials eluted substances over a period of 5 weeks. Identified substances included bisphenol A (BPA), triethylene glycol dimethacrylate (TEGDMA) and urethane dimethacrylate (UDMA). BPA eluted from Transbond™ Plus, XT, LR and BrackFix®. The cumulated mean values after 35 days ranged from 16.04 to 64.83 ppm, depending on the material. TEGDMA eluted with a mean of 688.61 ppm from Transbond™ LR. UDMA with a mean of 1682.00 ppm from Triad®Gel. For each material the highest concentrations of all these substances were found in the first elution period. Other substances that were not equivocally identified or of low concentration also eluted.

**Conclusion:**

Using the described method, it is possible to qualitatively and quantitatively determine the in vitro elution of monomers from orthodontic materials. The concentrations of the substances identified were below the current maximum recommended intake. However, a cumulative effect and low-dose effects should be considered for both patients and dental professionals, especially for young patients. Measures to reduce exposure patients and practitioners are suggested.

## Abstract (de)

**Ziel:**

Das übergeordnete Ziel dieser Untersuchung war die qualitative und quantitative Untersuchung gebräuchlicher kieferorthopädischer Materialien hinsichtlich ihrer Monomerfreisetzung. Studien haben erhebliche gesundheitliche Auswirkungen von Monomeren nachgewiesen, etwa ihr zytotoxisches, allergenes bzw. mutagenes Potenzial und endokrine Veränderungen. Diese In-vitro-Studie konzentriert sich vor allem auf 5 Kunststoffe, die in der Regel intraoral verarbeitet werden und langfristig dort verbleiben.

**Methoden:**

Untersucht wurde die Freisetzung von Monomeren aus Proben (7.5 mm × 1.5 mm), die bei Körpertemperatur (37 °C) für 30 min bis 5 Wochen in künstlichen Speichel getaucht wurden. Die dabei verwendete Methode ist konform mit DIN EN ISO 10993-13. Die 5 getesteten Materialien waren BrackFix® (Voco GmbH, Cuxhaven), Triad®Gel (DeguDent GmbH, Hanau) und Transbond™ XT, LR und Plus (3M Unitek, Monrovia/CA, USA). Alle Aliquots wurden mit Hochleistungsflüssigkeitschromatographie (HPLC) analysiert.

**Ergebnisse:**

Alle 5 analysierten Materialien eluierten Substanzen über einen Zeitraum von 5 Wochen. Zu den identifizierten Substanzen gehörten Bisphenol A (BPA), Triethylenglykol-Dimethacrylat (TEGDMA) und Urethan-Dimethacrylat (UDMA). BPA wurde aus Transbond™ Plus, XT, LR und BrackFix® eluiert. Die kumulierten Mittelwerte nach 35 Tagen reichten je nach Material von 16,04–64,83 ppm. TEGDMA eluierte mit einem Mittelwert von 688,61 ppm aus Transbond™ LR, UDMA mit einem Mittelwert von 1682,00 ppm aus Triad®Gel. Höchstwerte im Verhältnis von Menge zur Zeit der freigesetzten Substanzen wurden für jedes Material in der initialen Periode ermittelt. Weitere, nicht identifizierte sowie gering konzentrierte Substanzen wurden ebenfalls eluiert. Für jedes Material wurden die höchsten Konzentrationen all dieser Substanzen in der ersten Elutionsperiode gefunden. Andere Substanzen, die nicht eindeutig identifiziert wurden oder von geringer Konzentration waren, wurden ebenfalls eluiert.

**Schlussfolgerung:**

Mit der beschriebenen Methode ist es möglich, die In-vitro-Elution von Monomeren aus kieferorthopädischen Materialien qualitativ wie quantitativ zu bestimmen. Die Konzentrationen der identifizierten Substanzen lagen unterhalb der derzeit empfohlenen maximalen Aufnahmemenge. Dennoch sollten eine kumulative Wirkung und Niedrigdosiseffekte sowohl für Patienten, vor allem für junge Patienten, als auch für das zahnärztliche Personal in Erwägung gezogen werden. Maßnahmen zur Verringerung der Exposition von Patienten und Behandelnden werden vorgeschlagen.

## Introduction

An orthodontic treatment requires various materials for removable and fixed appliances. The period of time orthodontic appliances stay in the oral cavity ranges from minutes to several years, where environmental influences affect the materials’ durability and biodegradation. Important variables include thermal and pH value changes, enzymatic and bacterial activity, or mechanical alteration. For example, food, saliva, muscular activity and the stress from dimensional changes cause extreme conditions for any material.

Besides different metal alloys and ceramics, various resins with similar composition to dental fillings are frequently used for multiple appliances and purposes in treatments. In orthodontics, acrylic monomers are for example found in aligners, removable appliances such as functional appliances or removable retainers or as bonding material between teeth and brackets or lingual retainers.

The chemical composition of dental and orthodontic resins and polymers can be divided into three major components. Namely, an organic phase, a disperse phase with inorganic fillers determining mechanical and physical properties, and a bonding phase or coupling agent. Characteristics like viscosity or shrinkage during polymerization differ due to this composition. The organic phase contains monomers, oligomers, initiators, inhibitors and other additives. Main representatives of the monomers are substances with high molecular weight for less shrinkage like bis-GMA (bisphenol A glycidyl dimethacrylate) and UDMA (urethane dimethacrylate) or low-molecular-weight substances for improved flowability such as HEMA (2-hydroxyethyl methacrylate) and TEGDMA (triethylene glycol dimethacrylate) [2, 22, 40].

Several articles have investigated common monomers in orthodontics. Especially BPA (bisphenol A), used as a starter ingredient for bis-GMA or bis-EMA (ethoxylated bisphenol A dimethacrylate), but also others like TEGDMA and UDMA were in the focus of researchers [17, 19, 34, 36, 45, 48].

Since these polymers never reach a full degree of conversion after photo- or self-curing polymerization, residual and leaching monomers from methacrylate-based restorative and orthodontic materials are seen as a result [5, 21, 23]. These substances have been found in different tissues and fluids [3, 4, 35, 37, 46, 54], causing allergic [18, 28], teratogenic [46, 49], cytotoxic [7, 12, 16, 20], mutagenic [14, 25, 41], neurotoxic [61], endocrine effects [38, 57], fertility disorders [29, 59] and DNA damage [47, 60] or epigenetic programming [44]. Furthermore, early exposure to monomers is suspected of causing molar incisor hypomineralization (MIH) and having an impact on the psychosocial health of children [24, 32, 33].

The European Food Safety Authority (EFSA) and the U.S. Food and Drug Administration (FDA) reacted to the scientific findings and set limits to a maximal daily intake of BPA in the past [13]. In 2015, the EFSA lowered the threshold from 50 to 4 µg/kg body weight/day. Since 2011 there has been a ban on BPA in baby bottles in the EU [8]; in 2015 a complete ban on BPA from all food packaging was introduced in France, while other European countries added similar restrictions [6]. However, despite the many studies mentioned above which found local, systemic and synergistic effects of TEGDMA and UDMA, even in low doses, no thresholds exist, yet. Discussions about health threats and environmental issues from polymers are the reason for restrictions and an ongoing controversy. Against this background it is also the responsibility of every orthodontist to use and seek alternatives to minimize potentially hazardous uptake of these substances from orthodontic materials.

In the present study we tested five frequently used acrylic based orthodontic light-curing resins with different indications: BrackFix® (BrackFix; Voco GmbH, Cuxhaven, Germany) and Transbond™ XT (Transbond™ XT; 3M Unitek, Monrovia, CA, USA) to bond metal and ceramic brackets to tooth surfaces, Transbond™ LR (Transbond™ LR; 3M Unitek, Monrovia, CA, USA) for bonding fixed lingual retainers, Transbond™ Plus (Transbond™ Plus; 3M Unitek, Monrovia, CA, USA) a band adhesive and Triad®Gel (TriadGel; DeguDent GmbH, Hanau, Germany) as a multipurpose material.

It was the aim of this in vitro investigation to study leakage of BPA, Bis-GMA, CQ (camphorquinone), HEMA, HQ (hydroquinone), MMA (methyl-methacrylate), TEGDMA, and UDMA using artificial saliva at body temperature as elution medium, analyzed by high performance liquid chromatography (HPLC).

## Materials and methods

The five tested orthodontic materials and their compositions, instructions and test parameters are listed in Table 1.

**Table 1:** Orthodontic materials and their compositions, instructions and test parameters Kieferorthopädische Materialien: Bestandteile, Anwendungshinweise und Prüfparameter

Product and manufacturer	Composition from safety data sheets (if given: CAS No.)	Polymerization instructions	Test parameters
*Triad®Gel* DeguDent GmbH, Hanau, Germany	Urethane dimethacrylate (UDMA) Silicon dioxide Pigments Initiators Stabilizers	30 s in Triad® 2000 Light Curing Unit (Dentsply International, York, PA, USA)	Light intensity/time: 1600 mW/cm^2^ for 30 s Curing light distance: 2 mm Mean sample weight: 0.071 g
*Transbond™ Plus* 3M Unitek, Monrovia, CA, USA	Silane-treated glass Glycerol 1,3-dimethacrylate (1830-78-0) Citric acid dimethacrylate oligomer Silane-treated silica (248596-91-0) Diphenyliodonium hexafluorophosphate (58109-40-3)	Light intensity/time: 1600 mW/cm^2^ for 30 s Curing light distance: 1–2 mm	Light intensity/time: 1600 mW/cm^2^ for 30 s Curing light distance: 1 mm Mean sample weight: 0.129 g
*Transbond™ XT* 3M Unitek, Monrovia, CA, USA	Silane-treated quartz (10042-78-6) Bisphenol A diglycidyl ether dimethacrylate (bis-GMA) (1565-94-2) Bisphenol A bis(2-hydrocyethyl ether) dimethacrylate (24448-20-2) Silane-treated silica (68611-44-9) Diphenyliodonium hexafluorophosphate (5810940-3)	Light intensity/time: 1600 mW/cm^2^ for 3 s Curing light distance: 2–3 mm	Light intensity/time 1600 mW/cm^2^ for 3 s Curing light distance: 2 mm Mean sample weight: 0.133 g
*Transbond™ LR* 3M Unitek, Monrovia, CA, USA	2‑Propenoic acid, 2‑methyl-, 3‑(trimethoxysilyl)propyl ester, reaction products with quartz (100402-78-6) Bisphenol A diglycidyl ether dimethacrylate (bis-GMA) (1565-94-2) Triethylene glycol dimethacrylate (TEGDMA) (109-16-0) Dichlorodimethylsilane reaction product with silica (68611-44-9) Ethyl-4-dimethylaminobenzoate (EDB) (10287-53-3) Diphenyliodonium hexafluorophosphate (58109-40-3)	Light intensity/time: 1600 mW/cm^2^ for 10 s Curing light distance: not given	Light intensity/time 1600 mW/cm^2^ for 10 s Curing light distance: 2 mm Mean sample weight: 0.140 g
*BrackFix®* VOCO GmbH, Cuxhaven, Germany	Bis-GMA (1565-94-2) Bis-EMA (41637-38-1)	Light intensity/time: min. 1000 mW/cm^2^ for 20 s Curing light distance: 1–2 mm	Light intensity/time: 1200 mW/cm^2^ for 20 s Curing light distance: 1 mm Mean sample weight: 0.135 g

Polyoxymethylene (POM) rings between two glass microscope slides were used to create ten identical disc-shaped samples (7 mm diameter, 1.5 mm thick, 76.97 mm^2^ exposed surface area, 57.73 mm^3^ volume) each of BrackFix®, Triad® Gel, Transbond™ XT, Transbond™ LR and Transbond™ Plus.

Every material was light-cured with a LED light-curing unit (VALO C0 1516 LED, Ultradent Products Inc., South Jordan, UT, USA), following the instructions provided by the manufacturers regarding light intensity, wavelength and polymerization time. Individually manufactured polymerization-stands for each material were used to keep the recommended distance from sample to curing light. The light unit was routinely tested by a photometer (Bluephase® Meter II; Ivoclar Vivadent AG, Schaan, Liechtenstein), the mean output was 1188 mW/cm^2^ (1200 mW/cm^2^ mode) and 1604 mW/cm^2^ (1600 mW/cm^2^ mode), measured through a microscope slide.

Each sample was weighed and afterwards immersed in new glass sample vials with tetrafluoroethylene-lined caps (20 mL, GL 18, Duran®, Schott AG, Mainz, Germany) to prevent contamination, containing 1.5 mL artificial saliva type Fusayama/Meyer (Pickering Laboratories, Inc., Mountain View, CA, USA). The POM ring was attached to a nylon string (diameter 0.16 mm, folia, Max Bringmann KG, Wendelstein, Germany) allowing the tested materials to move freely in the medium. The glass tubes were placed on an individually manufactured circular tube tray, rotating at 60 rpm by a shaker (3D Sunflower Mini Shaker, BioSan, Riga, Latvia) in an incubator (Heraeus, Hanau, Germany) at +37 °C, simulating intraoral conditions.

In accordance with DIN EN ISO 10993-13, specimens of 1 mL of the test tubes (artificial saliva, POM ring, sample, nylon string) and a control tube (artificial saliva, POM ring, nylon string) were collected 30 min, 3 h, 6 h, 24 h, 48 h, 72 h, 7 days, 14 days, 21 days, 28 days and 35 days after immersion (t1–t11). Each time, 1 mL of fresh artificial saliva was added to the glass tubes afterwards.

The specimens were stored at +4 °C in HPLC vials to prevent contamination or reactions with monomers or other substances. In addition, the experiments were performed in a laboratory protected from UV light. All aliquots were analyzed by high performance liquid chromatography (HPLC). The setup and settings are listed in Table 2. The monomers and calibration standards in methanol are summarized in Table 3. The detection of the monomers in the specimens was based on the retention time and the spectrum given by the calibration standards and the quantification by the area under the curve (AUC) of each sample and standard. Four monomer-standards were analyzed during each HPLC run. One in artificial saliva and another one in distilled water, each before and after measuring the specimen to identify possible differences in the retention time and peak appearance. To minimize error sources, all procedures and an individual control of every chromatogram were performed by the same investigator (first author), including baseline adaptions, the identification of split peaks, fronting, tailing or other deviations in the chromatograms.

**Table 2:** HPLC: setup and parameters HPLC: Aufbau und Parameter

	Model	Manufacturer
Controller	SCL-10A VP	Shimadzu, Kyoto, Japan
Pump A	LC-10AD VP	Shimadzu, Kyoto, Japan
Pump B	LC-10AD VP	Shimadzu, Kyoto, Japan
Diode array detector	SPD-M10A VP	Shimadzu, Kyoto, Japan
Autosampler	SIL-10A	Shimadzu, Kyoto, Japan
Column oven	CTO-10 AS VP	Shimadzu, Kyoto, Japan
Column	EC 125/2 Nucleosil 100‑5 C18 Nautilus	Macherey-Nagel, Düren, Germany
Degasser	Degasys DG-1210	UniFlows, Tokyo, Japan
Software	Class VP 4.7	Shimadzu, Kyoto, Japan
Settings	Mobile phase	C_2_H_3_N 90%, aqua dest. 10%
	Detection	200–340 nm
	Flowspeed	0.25 mL/min

*HPLC* high pressure liquid chromatography

**Table 3:** Monomers used in calibration standards Für die Kalibrierungsstandards verwendete Monomere

Substance	Abbreviation	CAS No.	Manufacturer
Hydroquinone	HQ	123-31‑9	Sigma-Aldrich^a^
2‑Hydroxyethyl methacrylate	HEMA	868-77‑9	Sigma-Aldrich^a^
Methyl methacrylate	MMA	80-62‑6	Sigma-Aldrich^a^
Camphorquinone	CQ	10373-78‑1	Sigma-Aldrich^a^
Triethylene glycol dimethacrylate	TEGDMA	109-16‑0	Sigma-Aldrich^a^
Bisphenol A (2,2-Bis(4-hydroxyphenyl)propane, 4,4′-isopropylidenediphenol)	BPA	80-05‑7	Sigma-Aldrich^a^
Diurethane dimethacrylate	UDMA	72869-86‑4	Sigma-Aldrich^a^
Bisphenol A glycerolate dimethacrylate	Bis-GMA	1565-94‑2	Merz Dental^b^

^a^Sigma-Aldrich, St. Louis, MO, USA

^b^Merz Dental GmbH, Lütjenburg, Germany

The obtained data for the BPA release from four materials was analyzed with a two-way analysis of variance (ANOVA) to evaluate the impact of time and different materials on the elution process. The *p* value of the interaction analysis has to be considered as exploratory due to the nature of the evaluation.

The following software were used for data processing and visualization: Class VP 4.7 (Shimadzu, Kyoto, Japan), Excel® for Mac 16.23 (Microsoft, Redmond, WA, USA), SPSS® Statistics 26 (IBM, Armonk, NY, USA) and OriginPro 2019 (OriginLab, Northampton, MA, USA).

## Results

**Fig. 1:**
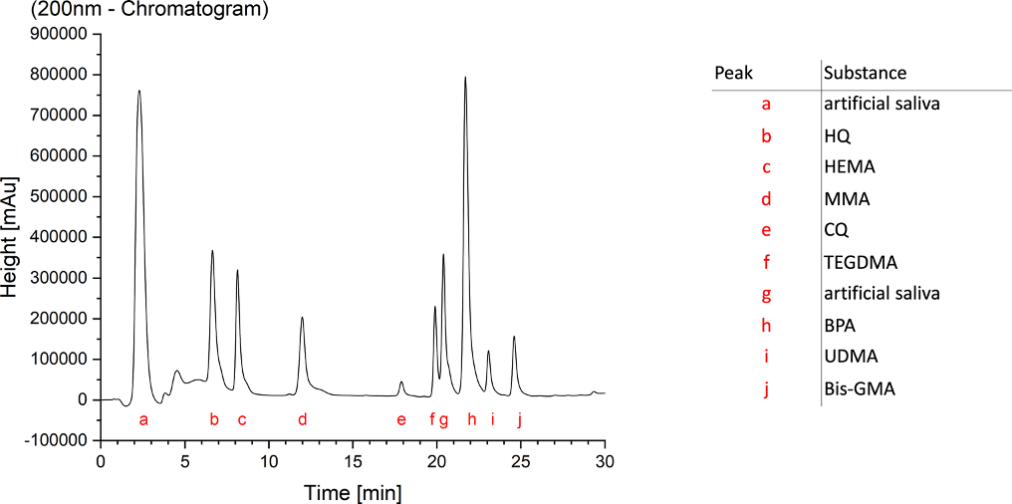
Chromatogram of a monomer standard in artificial saliva with assignment of the peaks. Monomer abbreviations provided in Table 3 Chromatogramm eines Monomerstandards in künstlichem Speichel mit Zuweisung der Peaks. Abkürzungen für Monomere s. Tab. 3

The final chromatograms made an analysis of the standards, control group and specimens possible (Fig. 1). All five materials released monomers and other not uniquely identifiable substances (Table 4). Samples with a 76.97 mm^2^ surface area and 57.73 mm^3^ volume were immersed in 1.5 mL. The mean weight of the samples was 0.071 g (Triad® Gel), 0.129 g (Transbond™ Plus), 0.133 g (Transbond™ XT), 0.14 g (Transbond™ LR) and 0.135 g (BrackFix®). The minimum detection threshold for the identification and quantification of peaks in the chromatograms was set to the area under curve (AUC) value of 100,000 in ClassVP 4.7.

**Table 4:** Results: Eluted substances from each material (ppm [μg/1 g material]) from t0–t11 and cumulated values after 35 days (d) Ergebnisse: Aus jedem Material eluierte Substanzen (ppm [μg/1 g Material]) von t0–t11 und kumulierte Mengen nach 35 Tagen

*Material*	*Eluted Substances*	*t1 (30* *min)*			*t2 (3* *h)*			*t3 (6* *h)*			*t4 (24* *h)*			*t5 (48* *h)*			*t6 (72* *h)*		
		*Mean*	*Median*	*±SD*	*Mean*	*Median*	*±SD*	*Mean*	*Median*	*±SD*	*Mean*	*Median*	*±SD*	*Mean*	*Median*	*±SD*	*Mean*	*Median*	*±SD*
Triad®Gel	UDMA	151.19	144.42	24.92	189.08	178.60	43.83	146.28	147.24	34.04	229.41	227.93	30.51	169.19	171.11	44.29	152.23	148.60	36.81
Transbond™ Plus	BPA	3.29	3.42	0.67	2.07	2.07	0.40	1.09	1.05	0.36	1.75	1.82	0.43	1.09	1.15	0.60	1.23	1.10	0.54
Transbond™ XT	BPA	3.91	3.68	0.65	6.19	6.25	1.02	4.90	5.06	0.84	9.37	9.08	1.31	7.10	6.94	1.00	5.83	5.71	0.49
Transbond™ LR	TEGDMA	208.87	201.57	87.75	139.10	120.93	71.12	62.49	59.94	21.76	111.52	110.80	28.79	39.57	45.43	21.51	23.76	26.72	5.32
	BPA	2.45	2.94	1.29	2.19	2.70	1.22	1.60	1.75	0.97	2.38	3.06	1.30	1.64	1.84	0.91	1.25	1.51	0.72
BrackFix®	BPA	1.51	1.39	0.45	1.38	1.44	0.27	1.64	1.56	0.50	2.74	2.78	0.67	2.08	2.14	0.42	1.54	1.59	0.44
*Material*	*Eluted Substances*	*t7 (7* *d)*			*t8 (14* *d)*			*t9 (21* *d)*			*t10 (28* *d)*			*t11 (35* *d)*			*cumulative (t1–t11)*		
		*Mean*	*Median*	*±SD*	*Mean*	*Median*	*±SD*	*Mean*	*Median*	*±SD*	*Mean*	*Median*	*±SD*	*Mean*	*Median*	*±SD*	*Mean*	*Median*	*±SD*
Triad®Gel	UDMA	169.87	185.24	43.21	141.76	148.16	41.57	129.47	129.65	36.85	110.00	114.48	22.34	99.54	101.96	20.35	1682.00	1764.07	262.07
Transbond™ Plus	BPA	2.03	2.36	0.80	1.10	1.05	0.23	1.07	0.94	0.47	0.79	0.83	0.26	0.73	0.80	0.23	16.04	15.69	1.84
Transbond™ XT	BPA	7.95	8.48	1.43	5.91	6.05	0.59	5.30	5.46	1.49	4.22	4.12	0.65	4.14	4.53	0.97	64.83	66.56	5.58
Transbond™ LR	TEGDMA	37.45	32.32	19.33	28.66	30.85	11.66	16.99	14.40	8.50	11.88	11.49	5.51	8.32	8.06	3.43	688.61	639.85	185.40
	BPA	1.75	1.90	1.02	1.53	1.75	0.80	1.49	1.79	0.81	1.09	1.38	0.58	1.10	1.31	0.59	18.46	23.27	9.49
BrackFix®	BPA	1.93	1.99	0.38	1.87	1.81	0.57	1.55	1.57	0.36	1.60	1.66	0.43	1.57	1.60	0.25	19.44	19.72	1.91

*SD* standard deviation, *BPA* bisphenol A, *TEGDMA* triethylene glycol dimethacrylate, *UDMA* urethane dimethacrylate

**Fig. 2:**
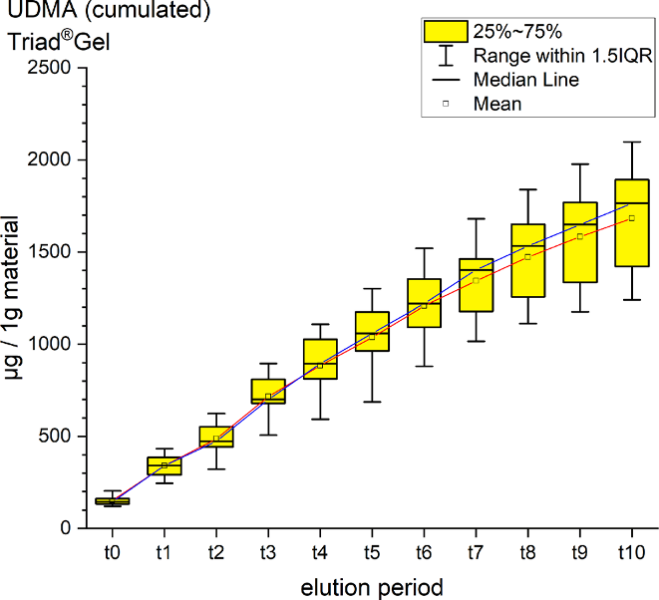
Cumulated quantity of eluted urethane dimethacrylate (UDMA [μg]) from 1 g Triad®Gel. The *red line* indicates the progression of the mean values; the *blue line* of the median values of each measurement point from t0–t11. *IQR* interquartile range Kumulierte Menge von eluiertem UDMA (Urethan-Dimethacrylat [μg]) aus 1 g Triad®Gel. Die *rote Kurve* stellt die Entwicklung der errechneten Mittelwerte dar, die *blaue Kurve* die der Mediane über den Untersuchungszeitraum (t0–t11). *IQR* Interquartilbereich

**Fig. 3:**
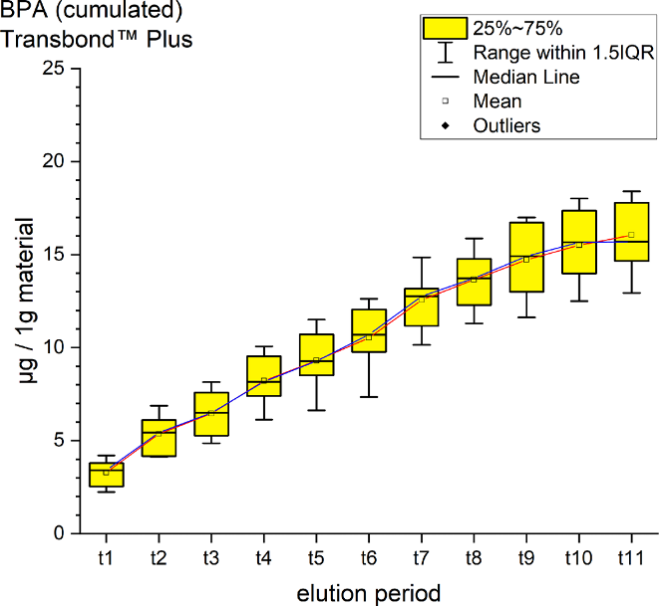
Cumulated quantity of eluted bisphenol A (BPA; [μg]) from 1 g Transbond™ Plus. The *red line* indicates the progression of the mean values from t0–t11; the *blue line* of the median. *IQR* interquartile range Kumulierte Menge von eluiertem BPA (Bisphenol A; [μg]) aus 1 g Transbond™ Plus. Die *rote Kurve* stellt die Entwicklung der errechneten Mittelwerte über den Untersuchungszeitraum (t0–t11) dar, die *blaue Kurve* die der Mediane. *IQR* Interquartilbereich

**Fig. 4:**
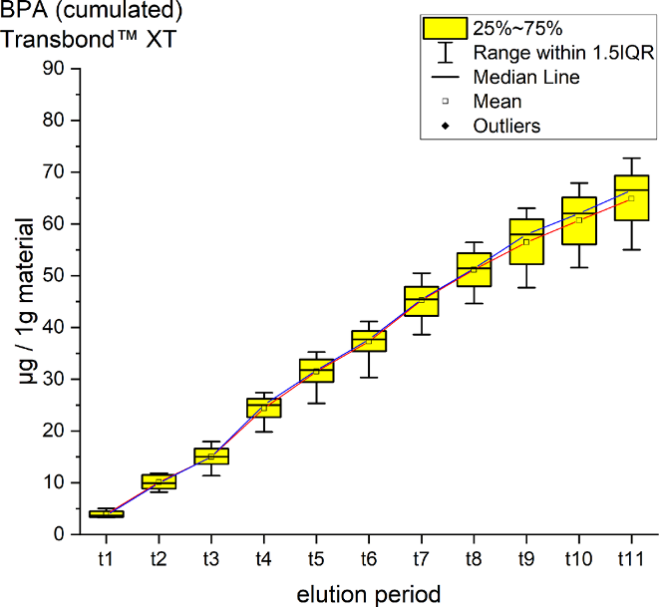
Cumulated quantity of eluted bisphenol A (BPA; [μg]) from 1 g Transbond™ XT. The *red line* indicates the progression of the mean values from t0–t11; the *blue line* of the median. *IQR* interquartile range Kumulierte Menge von eluiertem BPA [μg] aus 1 g Transbond™ XT. Die *rote Kurve* stellt die Entwicklung der errechneten Mittelwerte über den Untersuchungszeitraum (t0–t11) dar, die *blaue Kurve* die der Mediane. *IQR* Interquartilbereich

**Fig. 5:**
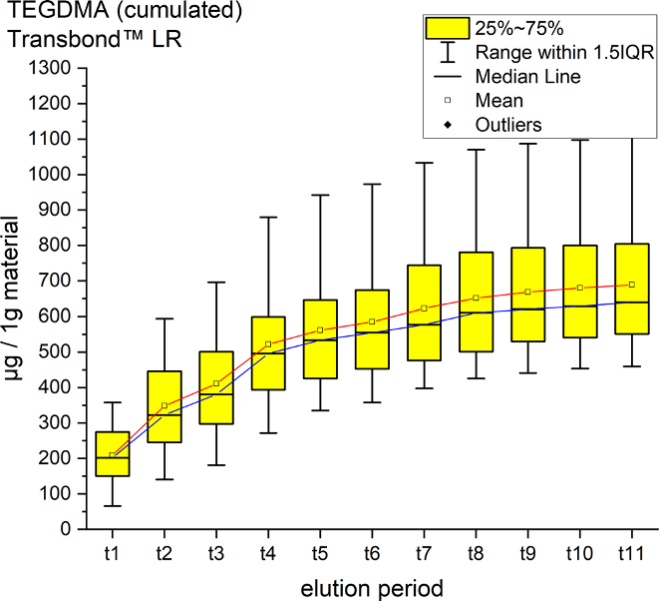
Cumulated quantity of eluted triethylene glycol dimethacrylate (TEGDMA; [μg]) from 1 g Transbond™ LR. The *red line* indicates the progression of the mean values from t0–t11; the *blue line* of the median. *IQR* interquartile range Kumulierte Menge von eluiertem TEGDMA (Triethylenglykol-Dimethacrylat; [μg]) aus 1 g Transbond™ LR. Die *rote Kurve* stellt die Entwicklung der errechneten Mittelwerte über den Untersuchungszeitraum (t0–t11) dar, die *blaue Kurve* die der Mediane. *IQR* Interquartilbereich

**Fig. 6:**
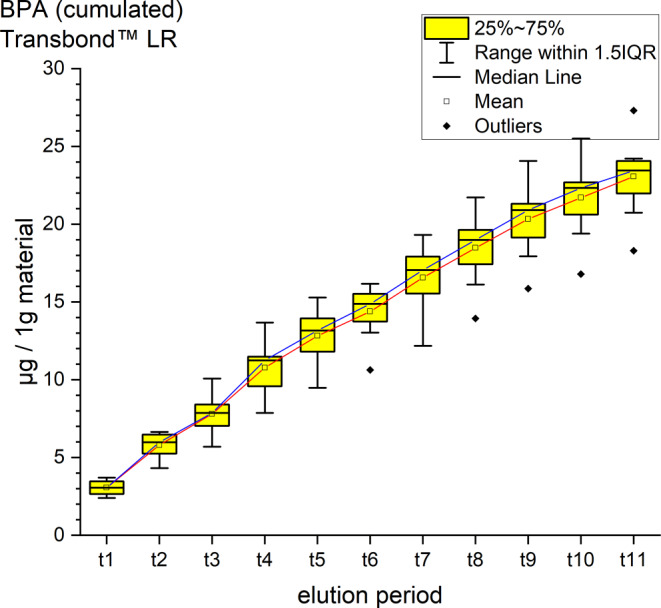
Cumulated quantity of eluted bisphenol A (BPA; [μg]) from 1 g Transbond™ LR. The *red line* represents the progression of the mean values from t0–t11, the *blue line* of the median. *IQR* interquartile range Kumulierte Menge von eluiertem BPA (Bisphenol A; [μg]) aus 1 g Transbond™ LR. Die *rote Kurve* stellt die Entwicklung der errechneten Mittelwerte über den Untersuchungszeitraum (t0–t11) dar, die *blaue Kurve* die der Mediane. *IQR* Interquartilbereich

**Fig. 7:**
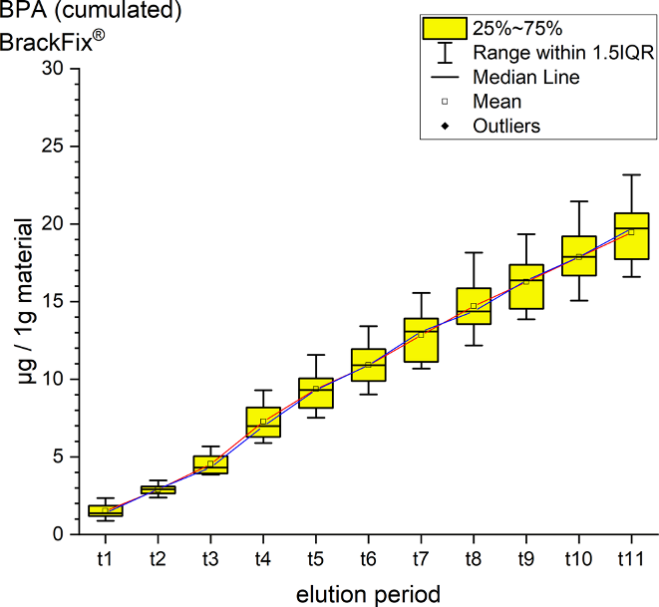
Cumulated quantity of eluted bisphenol A (BPA; [μg]) from 1 g BrackFix®. The *red line* represents the progression of the mean values from t0–t11, the *blue line* of the median. *IQR* interquartile range Kumulierte Menge von eluiertem BPA (Bisphenol A; [μg]) aus 1 g BrackFix®. Die *rote Kurve* stellt die Entwicklung der errechneten Mittelwerte über den Untersuchungszeitraum (t0–t11) dar, die *blaue Kurve* die der Mediane. *IQR* Interquartilbereich

It was possible to identify and quantify BPA, UDMA and TEGDMA by their retention time and spectrum (Table 4, Figs. 2, 3, 4, 5, 6 and 7). Other peaks were found, either below the threshold or not matching with monomers used in the standards for calibration.

Triad®Gel eluted UDMA with a cumulative mean of 1682.00 ± 262.07 ppm after 35 days (t0–t11). It is the highest cumulative mean of a substance detected in the study. The highest elution occurred between t3 and t4 (6–24 h after immersion) with a mean of 229.41 ppm, while the lowest value was observed between 28 and 35 days (t10–t11) with a mean of 99.54 ppm (Fig. 2). No significant amounts of other unknown peaks were found in the chromatograms.

Transbond™ Plus eluted BPA and two not uniquely identified substances. A cumulative mean of 14.44 ± 1.84 ppm BPA after 35 days was detected. A maximum peak with a mean of 9.37 ppm at t1 (after 30 min) and a minimum mean of 0.73 ppm at t11 (Fig. 3). The unidentified peaks showed retention times similar to the retention times in our standards of hydroquinone and camphorquinone. Both, just as BPA, are not mentioned in the safety data sheets provided by the manufacturer.

Transbond™ XT eluted a cumulative mean of64.83 ± 5.58 ppm BPA after 35 days, a maximum mean of 9.37 ppm at t4, a minimum (3.91 ppm) at t1 (Fig. 4). It is the highest cumulative mean of BPA from all the tested materials. Smaller peaks of bis-GMA below the threshold were also identified.

Transbond™ LR eluted TEGDMA and BPA. TEGDMA eluted the second highest cumulative mean of the study with 688.61 ± 185.40 ppm after 35 days, an elution maximum with a mean of 208.87 after 30 min (t1) and a minimum of 8.32 ppm at t11 (Fig. 5). The cumulative mean of BPA was 18.46 ± 9.49 ppm after the 35 days, the maximum elution was measured at t1 with 2.45 ppm and a minimum of 1.09 ppm at t10 (Fig. 6).

BrackFix® eluted 19.44 ± 1.91 ppm of BPA in 35 days. The maximum mean was at t4 with 2.74 ppm, the minimum at t2 was 1.38 ppm (Fig. 7). Smaller peaks of bis-GMA under the set threshold were also identified.

**Fig. 8:**
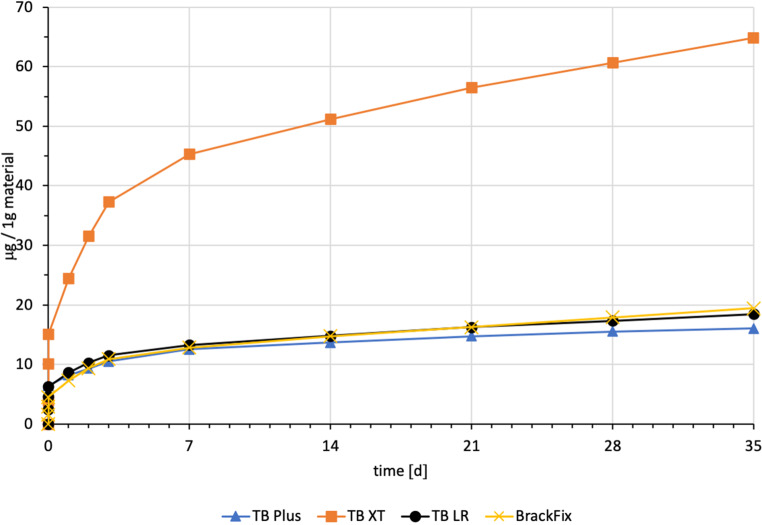
Comparison of the cumulated quantities of eluted bisphenol A (BPA, [μg]) from 1 g Transbond™ Plus, Transbond™ XT, Transbond™ LR, BrackFix® over 35 days Gegenüberstellung der kumulierten Mengen von eluiertem BPA (Bisphenol A; [μg]) aus 1 g Transbond™ Plus, Transbond™ XT, Transbond™ LR, BrackFix® im zeitlichen Verlauf über 35 Tage

**Fig. 9:**
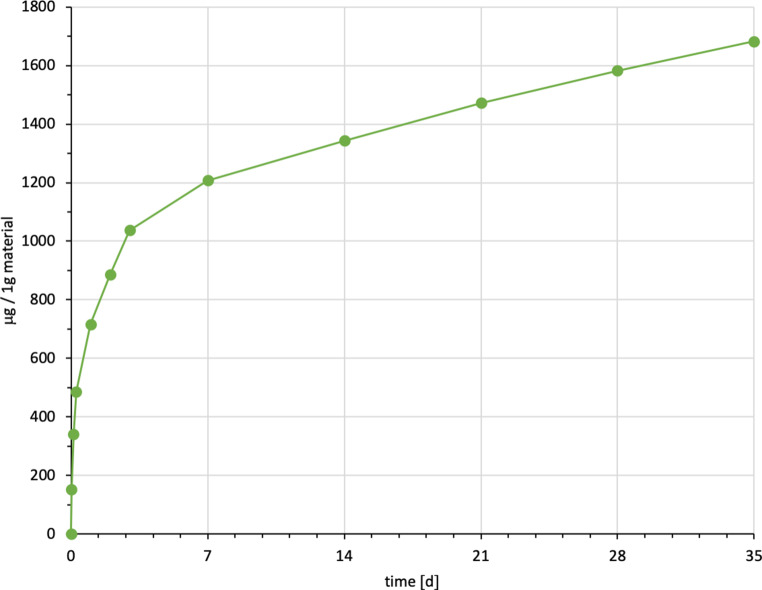
Cumulated quantity (mean) of eluted urethane dimethacrylate (UDMA; [μg]) from 1 g Triad®Gel over 35 days (d) Kumulierte Mengen (Mittelwerte) von eluiertem UDMA (Urethan-Dimethacrylat; [μg]) aus 1 g Triad®Gel im zeitlichen Verlauf über 35 Tage

**Fig. 10:**
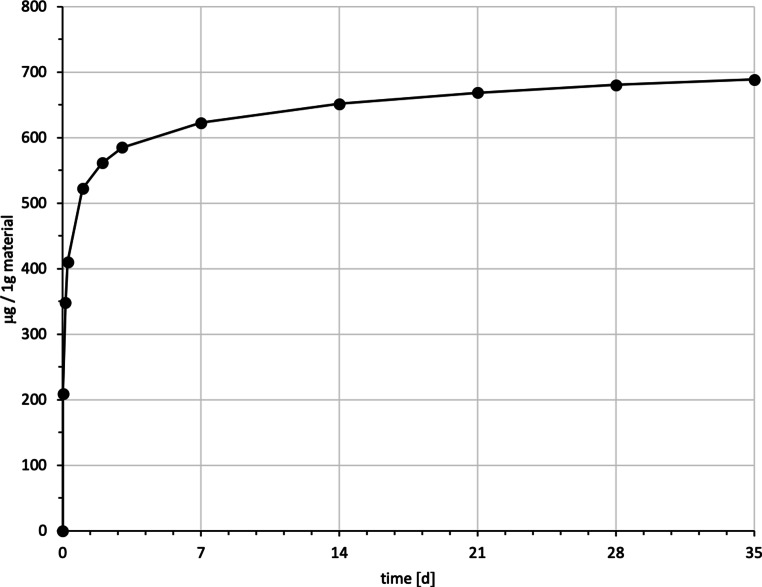
Cumulated quantity (mean) of eluted triethylene glycol dimethacrylate (TEGDMA; [μg]) from 1 g Transbond™ LR over 35 days (d) Kumulierte Mengen (Mittelwerte) von eluiertem TEGDMA (Triethylenglykol-Dimethacrylat; [μg]) aus 1 g Transbond™ LR im zeitlichen Verlauf über 35 Tage

For every tested material and identified substance, the average amount of the eluted substances in ppm per hour showed a maximum for the values of t1 (0–30 min) and a minimum at t11 (28–35 days interval; Figs. 8, 9 and 10).

For BPA the time-dependent curves (Fig. 8) of the four materials were different (interaction *p* value from 2‑way ANOVA < 0.001). Where Transbond™ Plus, Transbond™ LR and BrackFix® showed very similar low elusion rates (maximum cumulated mean elusion after 35 days (t11) from 14.44 ppm to 19.44 ppm, see above), Transbond™ XT showed a substantially higher elusion (64.83 ppm). The cumulated elution of BPA from Transbond™ XT after 35 days (t11) was approximately four times higher than from the other three materials.

## Discussion

This HPLC study identified and quantified the release of three monomers (BPA, UDMA and TEGDMA) from five different orthodontic materials (BrackFix®, Triad® Gel, Transbond™ XT, Transbond™ LR, Transbond™ Plus) eluted in artificial saliva over 5 weeks.

Various substances listed in the safety data sheets (SDS) and also not uniquely identified or not mentioned eluted substances have been detected. Every identified substance, besides BPA leaching from Transbond™ Plus, is listed in the SDS of the tested materials. The eluted and detected BPA, (or possible BPA derivatives as persecutors of bis-GMA and bis-EMA), is possibly a degradation product of larger substances, or its match in composition is too small to be mentioned in the SDS [27, 30]. Other studies also found substances not mentioned in the SDS [1]. Monomers of smaller molecular weight, such as TEGDMA or BPA are more likely and faster to leach from dental materials than monomers of higher molecular weight (e.g., bis-GMA, Figs. 8, 9 and 10; [52]). In addition, smaller peaks below the threshold with the same retention time as bis-GMA appeared in the chromatograms of Transbond™ Plus and BrackFix® aliquots.

Other substances were released but could not be identified. The identity of those can be assumed by comparing their characteristics in the chromatograms with other monomers used in the analyzed standards. For Transbond™ Plus, besides low concentrations of BPA, two unidentified peaks can be seen. The characteristics of the peaks do show a similar, but not the exact and constant retention time as hydroquinone (HQ) and camphorquinone (CQ) used in the standards. Furthermore, the spectrum of the peaks differs as well to HQ and CQ. The additional substances (HQ and CQ) and others in the standards are often found in orthodontic and dental resins. They are for example used as photoinitiators, inhibitors and stabilizers or as residues of the raw materials. Even if not mentioned in the SDS, especially gas chromatography/mass spectrometry (GC/MS) studies have shown a release of CQ [1, 39, 50]. The SDS of Transbond™ Plus gives information about two substances (glycerol 1,3-dimethacrylate, diphenyliodonium hexafluorophosphate) not used in the standard. The two substances are suspected of causing the unidentified peaks in the chromatograms of Transbond™ Plus aliquots.

Comparing the materials and the measured amounts of the identified substances to each other, Triad®Gel eluted the most with a mean of 1682 ppm of UDMA after 35 days. Followed by Transbond™ LR with a cumulative mean of 688.61 ppm TEGDMA. For both substances, there are no limits published for a maximum daily intake by authorities.

BPA was found to eluate from four materials. The highest cumulative mean showed Transbond™ XT (64.83 ppm) after 35 days, followed by BrackFix® (19.44 ppm), Transbond™ LR (18.46 ppm) and Transbond™ Plus (14.44 ppm). Focusing on the elution process of BPA over time, three materials (Transbond™ LR, Transbond™ Plus, BrackFix®) showed similar characteristics in the graph. Only Transbond™ XT showed a major deviation compared to the others with higher concentrations and an extended elution process (Fig. 8). The already mentioned degradation process of larger molecular structures with time, different compositions of the materials or the unlikely case of another unknown substance with similar characteristics in the HPLC chromatogram leading to a larger peak with the retention time of BPA could explain this observation.

When it comes to the range of indications of the materials, both Transbond™ XT and BrackFix® are used for bracket bonding. Based on the results of our study BrackFix® should be preferred, provided that other material characteristics like sufficient tensile and shear strength are similar.

Comparing the results with other studies, it is important to point out the different approaches, which make comparisons difficult. The elution of material samples in artificial saliva at +37 °C aims at simulating intraoral conditions. However, the experimental setup still does not include possible impacts of bacterial and salivary enzymes in the human oral flora, pH and thermal changes [30]. Kloukos et al. [26] compared other studies in the field of interest, with the result that most authors used different methods. Different analyzing methods like liquid chromatography coupled with mass spectrometry (LC/MS) or gas chromatography (GC) coupled with MS show major differences in the results of the studies. Different elution media, such as water, ethanol or combinations are common. Ethanol is known to accelerate the aging of polymers [10], but it is questionable whether ethanol creates a comparable aging process as if the materials are immersed in saliva. Regarding the period of elution, we tried to create realistic conditions by expanding our study to a time period of 5weeks to extend the test period. The size, surface and weight of the specimens in perspective with the immersion medium and the time of elution are also factors that have to be taken into consideration when comparing different studies. In vivo, microfractures at the peripheral margins of orthodontic brackets and bands or within adhesives and a lower degree of conversion of the resins under brackets and bands could be another source of higher leakage of monomers. On the other side, the exposed surface of the resins to the oral cavity is most likely smaller than in our study, since resins for bracket bonding (BrackFix®, Trandbond™ XT) or cementing bands (Transbond™ Plus) are mostly covered by enamel, bands or bracket bases [10]. According to a review by Van Landuyt et al. the release of monomers depends on the exposed surface of samples, but there is no significant correlation with the volume [53].

Thus, it is difficult to determine the actual time of exposure, quantity, volume and surface area of the different materials used in vivo. Different numbers of teeth per patient, various appliances, the modification of these, the process of band or bracket bonding, re- and debonding or the removal of excess material are just a few potential variables. It is reasonable to conclude that the tested amount of the different materials in this study are far lower than the actual amount emerging during orthodontic treatment.

In general, we confirm the statement of most HPLC studies testing Transbond™ XT leakage of BPA [43, 51]. Only Malkiewicz et al. diverged from our study stating that there is no release of BPA from Transbond™ XT [30]. GC/MS studies support overall the results with the elution of BPA from Transbond™ XT [11, 27]. In addition, Transbond™ LR also eluted TEGDMA in our setup which is the first HPLC study showing that effect [39].

The applied method is an in vitro model attempting to replicate oral conditions based on the recommendations for a standardization by Kloukos et al. [26]. In combination with HPLC, this is a valid and simple method to identify and quantify different monomers released from orthodontic resins [51]. However, HPLC coupled with a mass spectrometry (MS) or a pre-analytical purification of aliquots might have identified further substances [31, 42]. However, adding more substances to the standards also expands the possibility to identify additional substances with the existing method. We analyzed new and previously tested materials with this method and compared our results with other studies [26].

Deviations in the appearance of the peaks in the chromatograms were found, resulting in variances of the area under the curve values and the relating calculated concentration of leached monomers in the aliquots. These anomalies might be the result of a pollution of the HPLC columns after several analytical runs, leading to split peaks, peak covering, fronting, broadening or tailing [58].

Possible disturbing factors which lead to the pollution of the columns are different ingredients of the artificial saliva like salts, unintentionally added smaller particles from the used materials or laboratory devices in the aliquots or debris from HPLC tubes. Major improvements in the appearance of the peaks in the chromatogram, such as less fronting or tailing and less split peaks or other signs for column pollution were achieved by the replacement of pre- and main columns during our research. In this study, the precolumns have been replaced three times, the main column once. For perfect conditions, the HPLC columns should be replaced, calibrated with standards and the system rinsed and purged regularly. Aliquots can be purified for even better conditions.

Additional disturbing factors which may lead to a wider spread of the results should be discussed and eliminated. For example, to prevent thermal changes, placing the HPLC in a room with a constant room temperature is recommended. In addition, reactions between the substances in the aliquots, due to ambient light or a different degree of conversation in the polymerization step as a result of differences in the curing light system, distance and time are possible [51].

To minimize sources of error, we standardized the process inter alia by using just one fully charged light-emitting diode device (LED) for every material, which is common in daily dental work and superior to a halogen light-curing unit [43]. Custom-made polymerization stands to keep the same distance were used. The experiments were performed in a laboratory protected from direct UV light. The time between pipetting aliquots and HPLC run was held constant throughout the study.

Regarding the medical point of view and potential health threats, the in vitro detected concentrations of BPA from the different materials in the aliquots of artificial saliva remained all below the threshold of 4 µg/kg body weight/day set by the EFSA in 2015 [8].

As a general premise it is important to minimize the exposure to any monomers. Any sources like food, packaging, daily polymers, paints, coatings, electric components and especially medical devices and appliances should be seen as part of the bigger picture [27]. Another reason for a potential higher exposure to monomers while undergoing orthodontic treatment is that volatile substances are not measured by this method. Possibilities of systematic intake other than through the oral environment include the respiratory and gastrointestinal tract or by physical contact [21]. Several in vitro and in vivo studies confirm our results that the concentrations of the eluted substance BPA are highest right after placement in the oral cavity or during the initial elution period [15, 37, 39]. In our study, every material and eluted substance showed the same effect: the highest concentrations were measured in the first period after bonding and the amount of eluted substances decreased with time (Figs. 8, 9 and 10). Thus, according to the eluted concentrations, a crucial period of time are the first 6 h (t0–t3) for substances with a low-molecular weight (BPA and TEGDMA, Figs. 8 and 10) and 72 h (t0–t6) for substances with a high-molecular weight (UDMA, Fig. 9). The lowest concentrations for every material were measured in the last period (t11). These characteristics are of major importance for the management of exposure during and right after the bonding and debonding process.

Teenagers are the major recipients of orthodontic treatment. This age is characterized by growth with several hormonal changes where endocrine-active substances like BPA might have an impact on development [27].

On the other hand, long-term effects for dental professionals should also be closely monitored to reduce potential harmful effects to their health. Further attempts to reduce monomer leakage and exposure to patients and dental professionals should be made. Common recommendations suggest dental dams while bonding, which is not practicable or can only be performed at disproportionate expense. Another suggestion is removing any excess adhesive before curing, generally to minimize the amount of material. The distance from material to the light-cure tip should be minimized to reach the highest degree of conversion as possible. The use of spray water and rinsing of the patients’ oral cavity multiple times with warm water after bonding and debonding is advisable. Also, suction devices should always be used to minimize aerosol while bonding and debonding. The use of alternative materials not containing any leaking monomers linked to health hazards, equipment for self-protection such as glasses, gloves and face masks are indicated [9, 26, 27].

In addition to existing regulations and recommendations, thresholds for other substances such as TEGDMA and UDMA should be considered by authorities. The impact of cumulative or low-dose effects over a long period should not be underestimated and should be taken into consideration [55, 56]. This will be of benefit for health providers, patients and the environment exposed to monomers.
